# Modelling Forward Osmosis Treatment of Automobile Wastewaters

**DOI:** 10.3390/membranes9090106

**Published:** 2019-08-22

**Authors:** Anita Haupt, Christian Marx, André Lerch

**Affiliations:** Chair of Process Engineering in Hydro Systems, Institute of Urban and Industrial Water Management, Technische Universität Dresden, 01062 Dresden, Germany

**Keywords:** automobile industry, forward osmosis, modelling, wastewater treatment

## Abstract

Forward osmosis (FO) has rarely been investigated as a treatment technology for industrial wastewaters. Within this study, common FO model equations were applied to simulate forward osmosis treatment of industrial wastewaters from the automobile industry. Three different models from literature were used and compared. Permeate and reverse solute flux modelling was implemented using MS Excel with a Generalized Reduced Gradient (GRG) Nonlinear Solver. For the industrial effluents, the unknown diffusion coefficients were calibrated and the influences of the membrane parameters were investigated. Experimental data was used to evaluate the models. It could be proven that common model equations can describe FO treatment of industrial effluents from the automobile industry. Even with few known solution properties, it was possible to determine permeate fluxes and draw conclusions about mass transport. However, the membrane parameters, which are apparently not solution independent and seem to differ for each industrial effluent, are critical values. Fouling was not included in the model equations although it is a crucial point in FO treatment of industrial wastewaters. But precisely for this reason, modelling is a good complement to laboratory experiments since the difference between the results allows conclusions to be drawn about fouling.

## 1. Introduction

In industries, increasing water scarcity combined with a high water demand as well as stricter laws for environmental protection has led to a growing awareness about efficient water usage [1]. Different technologies are used for wastewater treatment and water recycling, e.g., membrane filtration processes. Here, forward osmosis (FO) is a technology that has been investigated more and more within the last years [2,3,4,5,6].

**Figure 1:**
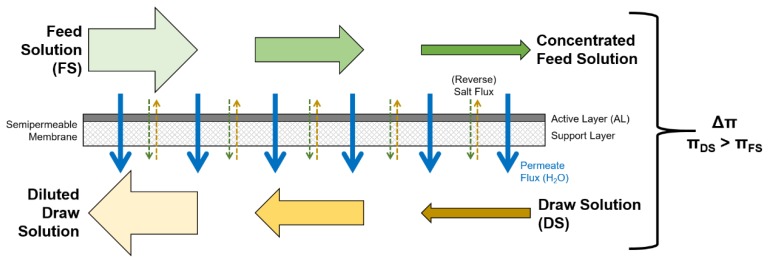
Forward osmosis (FO) process with membrane active layer facing towards the feed solution (FS) (active layer feed solution (ALFS) or FO-mode) **[6]**.

In FO, the osmotic pressure difference between the feed solution (FS) and the draw solution (DS) is the driving force, that makes water diffuse through a semipermeable membrane from FS into DS. This permeate flux dilutes the DS; the FS is concentrated (Figure 1). No physical pressure needs to be applied. In order to obtain pure water, the DS has to be regenerated in a separate treatment step. In many possible applications, an artificial DS needs to be used, such as salt solutions [7]. Here, the necessity of the DS regeneration steps is a main impact factor on economic efficiency [8,9]. 

The advantages of FO are low energy consumption, easy removable fouling layers, and treatable high salt concentrations [2,10,11,12]. The reverse solute flux through the membrane as well as concentration polarization are disadvantages [13,14] and are to be met by high performance FO membranes. FO membranes usually consist of a dense active layer (AL) and a porous support layer (SL). In the FO process, membranes can either be used with the AL facing the FS (ALFS orientation or FO mode) or with the AL facing the DS (ALDS orientation or PRO mode). An ALDS orientation enables higher permeate fluxes due to reduced concentration polarization within the SL [2]. However, due to the fact that the SL is more prone to fouling, the ALFS orientation is used more often [15]. FO membranes are characterised by three parameters: Water permeability A and solute permeability B, which relate to the active layer, and the structural parameter S, which relates to the support layer. These intrinsic parameters used to be determined by a combination of pressurized reverse osmosis tests and non-pressurized FO tests (RO–FO test) [16]. Lately, a methodology was suggested that utilizes only non-pressurized FO tests (FO-only test) [17]. If spacers are used in the FS and DS channels, their geometry also influences permeate flux and might be taken into consideration. The impact of spacers especially in spiral wound membrane modules has been studied for membrane filtration processes [18,19,20,21,22].

So far, FO has been applied for desalination and various water treatment processes [23,24,25,26,27]. However, few full-scale FO plants have been realized so far [8]. The application in industries has also been studied [6,28,29,30,31]. In these studies, mostly lab-scale experiments, which are time- and cost-consuming, were conducted to investigate FO performance. In order to get a rough estimation of FO performance for different application scenarios, it would be useful to simulate the FO process with a suitable model.

Several theoretical transport models have been proposed in the literature [16,32,33,34,35,36,37,38,39,40,41,42,43,44,45,46,47]. In 1981, Lee et al. introduced a model for pressure-retarded osmosis that described transport processes through an asymmetric membrane taking internal concentration polarisation (ICP) into account [32]. In 2006, McCutcheon et al. included not only ICP but also external concentration polarisation (ECP) on the selective membrane layer within their model [16]. In 2010, Philipp et al. reported about their model that described reverse solute flux (RSF) [37]. Shortly afterwards, Yip et al. and Tiraferri et al. published articles presenting FO models including RSF, ICP, and ECP on the selective membrane layer [44,46]. Here, Yip et al. considered an ALDS membrane orientation whereas Tiraferri et al. considered an ALFS membrane orientation. In 2015, Bui et al. then proposed a model that regarded RSF, ICP, and ECP on the selective layer as well as on the porous support layer [47].

However, so far these models have mostly been evaluated with experiments using NaCl or other salt solutions and deionized water only.

Within our research, three FO models from literature were partially adapted and integrated in MS Excel with a Generalized Reduced Gradient (GRG) Nonlinear Solver. FO performance was calculated for the treatment of different automobile wastewaters. The results from the FO experiments with these real wastewaters were used to evaluate the models.

## 2. Materials and Methods 

### 2.1. Lab-scale Experiments

Lab-scale experiments were conducted with four different wastewaters from an automobile manufacturing site as well as deionized water and 1 mol/L NaCl. The experimental set-up consisted of a flat-sheet membrane test cell with an active membrane area of 48 cm^2^. Flow channel dimensions in the test-cell were 1200 mm in length, 40 mm in width, and 0.86 mm in height on both sides of the membrane. During the experiment, FS and DS were circulated leading to a decreasing osmotic pressure difference overtime due to permeate flux. Thus, the observed permeate flux also decreased within the course of the experiment. Further decrease in permeate flux might be caused by membrane fouling. The detailed set-up has been described elsewhere [48]. 

CSM FO membranes from Toray Chemical Korea Inc. (Seoul, Korea) were used for all experiments. The manufacturers indicated that the standard permeate flux was 30 ± 5 L/(m^2^·h). A new membrane sample was used for every test series. 

The four different automobile wastewaters were cathodic dip painting rinsing water, cathodic dip painting wastewater, paint shop pre-treatment wastewater, and cooling tower circulation water. These waters were either used as FS or DS and combined with deionized water as FS, 1 mol/L NaCl as DS, or another effluent. FS and DS, osmotic pressure difference ∆π, and membrane orientation of the six experimental test series (A1–A6) and the performance tests (P) are given in Table 1. Further information can be found elsewhere [48].

Six test series with industrial wastewater were conducted using an ALFS membrane orientation, and one using an ALDS membrane orientation. The experimental procedure and the results of these test series are described in detail in another publication [48]. Within the experiments with industrial wastewaters, each test series consisted of three subsequent wastewater tests interrupted by a cleaning procedure and a membrane performance test. However, for the model evaluation, only the first wastewater test of each test series was used. One wastewater test lasted five hours.

Besides the wastewater tests, standard performance tests were conducted with deionized water as FS and 1 mol/L NaCl as DS. These tests lasted two hours and were, among others, performed before every test series when a new membrane sample was used. Thus, the experimental results of six performance tests were used for model evaluation.

### 2.2. Model Setup

In order to predict permeate flux *J_W_* and reverse solute flux *J_S_*, three different FO models were used for ALFS and ALDS membrane orientation (Table 2 and Table 3). These models were chosen because they are commonly used in FO modelling. 

Parameters A, B, and S are the water permeability in L/(m^2^·h·bar), the solute permeability in L/(m^2^·h) and the structural parameter of the membrane in m; π is the osmotic pressure in bar, *c* the molar concentration of NaCl in mol/L, and *D* the diffusion coefficient of the solution in m^2^/s; *k* in m/s describes the mass transfer coefficient from bulk solution to the membrane surface. Indexes FS and DS mean feed solution and draw solution, respectively.

Models I, II, and III all include internal concentration polarization but differ in the extent of external concentration polarization considered. Model I and Model II only consider external concentration polarization on the active layer side of the membrane. Model III combines internal concentration polarization and external concentration polarization on the active as well as the support layer of the membrane. The original Model I did not include the reverse solute flux and B was assumed to be zero [16]. Therefore, we used these results and added the concentrative ECP-term on the feed side, by keeping B ≠ 0. Furthermore, no equation for the RSF is offered, therefore the equation for the RSF of Model II was applied [46]. 

Membrane parameters A, B, and S were taken from literature as indicated in Table 4. Two different parameter sets, Par1 and Par2, were used: One that was determined by RO–FO tests and one that was determined by FO-only tests (see Table 4).

For modelling FO, it is necessary to consider the diffusion coefficient *D* of FS and DS. If the components and concentrations of FS and DS are known, the diffusion coefficient can be calculated [35,42]. However, industrial wastewaters like the automobile wastewaters are multi-component mixtures and analyzing all components would be very complex. Furthermore, the diffusion coefficient is prone to change during the FO experiment due to the concentration of FS and the dilution of DS, respectively, because of FS and DS circulation. For these reasons, it was decided to calibrate the diffusion coefficient ranging from 1^.^10^-11^ and 5^.^10^-9^ m^2^/s within the modelling procedure. This offers two advantages: Firstly, mixing of the solution within the support layer is covered by the calibrated diffusion coefficient; secondly, due to the unsure structural parameter *S*, the uncertainty and maybe errors can be compensated. Within the modelling procedure, flux calculation for one test run was repeated with a new diffusion coefficient several times. The diffusion coefficient was varied linearly by beginning at the lower boundary of the range and stepping upwards to the upper boundary of the calibration range.

The osmotic pressure π was determined from the osmolality, which was measured with a freezing-point microosmometer (Hermann Roebling Messtechnik, Germany), by applying Equation (1) [51]. Here, *c_osm_* is the osmolality, *T* the temperature and *R* the universal gas constant.

(1) $$\pi =c_{osm}\cdot R\cdot T$$

A virtual NaCl concentration β was determined by empirical Equations (2) and (3) using the measured electrical conductivity κ [48]. The molar concentration c was then calculated by multiplying with NaCl molecular weight. When pure NaCl solution or deionized water were the DS and FS, respectively, the exact concentration was used as input parameter for modelling. When automobile wastewaters were the DS and FS, only osmolality and electrical conductivity were used as input parameters in order to minimize analytical expense.

Low electrical conductivity κ < 1.0 mS/cm (β in g/L; κ in µS/cm)

(2) $$\beta =5.703\cdot 10^{-9}\;\cdot \;\kappa^{2}+4.9515\cdot 10^{-4}\;\cdot \;\kappa \;-\;6\cdot 10^{-4}$$

High electrical conductivity κ > 1.0 mS/cm (β in g/L; κ in mS/cm)

(3) $$\beta =1.4363\cdot 10^{-3}\;\cdot \;\kappa^{2}+0.5419\;\cdot \;\kappa \;+0.152$$

The mass transfer coefficient *k* was calculated using Equation (4) with diffusion coefficient *D*, Sherwood number *Sh*, and hydraulic diameter *d_h_* [16]. The Sherwood number *Sh* was calculated according to Equations (5) and (6) with Reynolds number *Re*, Schmidt number *Sc*, hydraulic diameter *d_h_,*, and channel length *L* [16].

(4) $$k=\frac{Sh\cdot D}{d_{h}}$$

(5) $$Sh\;=\;1.85{\left(Re\cdot Sc\cdot \frac{\;d_{h}}{L}\right)}^{0.33}\;\mathrm{laminar}\;\mathrm{flow}$$

(6) $$h=0.04\cdot Re^{0.75}\cdot Sc^{0.33}\;\mathrm{turbulent}\;\mathrm{flow}$$

The hydraulic diameter *d_h_* for our rectangular flow channel was calculated through the cross-section area *A_cs_* (width multiplied by height) and the wetted perimeter *l_u_* (doubled sum of width and height), as illustrated in Equation (7).

(7) $$d_{h}=\frac{4\cdot A_{cs}}{l_{u}}$$

The Schmidt number *Sc* is a dimensionless number, describing the relationship between the viscous diffusion (described by the dynamic viscosity η) and the mass diffusion, described by the diffusion coefficient *D* and the density ρ [52]. It was calculated by Equation (8).

(8) $$Sc=\frac{\eta }{\;D\cdot \varrho \;}$$

Equation (9) was used to calculate the Reynolds number with dynamic viscosity η, the density ρ, the fluid velocity *v*, and the cross-section area *A_cs_* [53]. Fluid velocity *v* was determined by dividing the known flowrate by cross-section area *A_cs_*. The density ρ in kg/m^3^ and the dynamic viscosity η in Pa^.^s were calculated by Equations (10) and (11), respectively, using the measured temperature ϑ in °C. These equations are empirically determined based on data published in literature and are valid for temperatures ranging from 0 to 30 °C [54,55].

(9) $$Re=\frac{\varrho \cdot v\cdot d_{h}}{\eta \cdot A_{cs}}$$

(10) $$\varrho =0.0000482484\cdot \vartheta^{3}-0.00819257\cdot \vartheta^{2}+0.0624602\cdot \vartheta +999.846$$

(11) $$\eta =0.0001\cdot \left(17.9098-0.6003\cdot \vartheta +0.01299\cdot \vartheta^{2}-0.000134\cdot \vartheta^{3}\right)$$

Permeate and reverse solute flux modelling was implemented using MS Excel from Microsoft Corporation (Redmond, WA, USA). The MS Excel workbook consisted of four worksheets: an introduction sheet as user-manual, an input data-sheet, a calibration-sheet, and a calculation sheet. All equations used are self-depending making it possible to solve the iteration with a Generalized Reduced Gradient (GRG) Nonlinear Solver from Frontline Systems Inc. (Incline Village, NV, USA) included in MS Excel. Precision for solving the iteration was set to 0.001. The temporal discretization for the model was chosen to be 1 min.

Furthermore, the following assumptions were made for the model:

- FS and DS behave like ideal solutions.
- The temperature is constant during the experiments.
- The permeate flux is directed from FS to DS.
- The reverse solute flux is directed from DS to FS.
- Membrane parameters A, B, and S are the same for all membrane samples.
- Membrane parameters A, B, and S are constant during the experiment.
- The diffusion coefficient D is constant during the experiment.
- Fouling does not occur.
- Chemical reactions do not occur.
- Spacers are not considered although they were used in the experiments.
- The fluxes axial across the membrane are constant; no local dependencies are assumed.
- The system is a steady-state system.

### 2.3. Model Evaluation

The introduced models were evaluated by comparing the simulated results with those from the experiments with real automobile wastewaters. Two evaluation parameters were used: The mean square error (MSE) and the Nash–Sutcliffe efficiency (NSE).

The MSE is a common way to describe model performance and is calculated according to Equation (12) with *J_mod_* and *J_exp_* being the modeled and the experimental flux at a certain time *t*. MSE values are strictly positive and the smaller they are, the better are the modeled results [56].

(12) $$MSE=\;\overset{N}{\underset{n=1}{\sum }}\;({J_{mod}^{t}-\;J_{exp}^{t})}^{2}$$

The Nash–Sutcliffe efficiency is used for evaluating the prediction of a hydraulic discharge and is commonly used for hydrological models [57]. The NSE is the normalized version of the MSE and calculated according to Equation (13). The NSE can range from -∞ to 1, whereby an efficiency of 1.0 indicates a perfect fit of modeled and experimental data. If the NSE is close to 0, the calculated model is as accurate as the mean value of the experimental data; whereby NSE values less than zero suggest that the experimental data is better than the calculated one. Good performing models should have an NSE between 0 and 1 [57].

(13) $$NSE=\;1-\;\frac{\sum_{n=1}^{N}\;{\left(J_{mod}^{t}-\;J_{exp}^{t}\right)}^{2}}{\sum_{n=1}^{N}\;{\left(J_{exp}^{t}-\;\bar{J_{exp}}\right)}^{2}}=1-\frac{MSE}{\sum_{n=1}^{N}\;{\left(J_{exp}^{t}-\;\bar{J_{exp}}\right)}^{2}}$$

## 3. Results and Discussion

### 3.1. Modelling the Permeate Flux for Standard Performance Tests with Deionized Water and 1 mol/L NaCl

As described in the previous chapter, standard membrane performance tests were performed with deionized water as FS and 1 mol/L NaCl as DS for 2 hours using ALFS mode. In the beginning of each test series, before the experiments with industrial effluents began, a performance test was conducted with a new membrane sample. Using the three models and two membrane parameter sets, the performance tests were modelled using diffusion coefficients ranging from 1.2 × 10^−9^ to 1.5 × 10^−9^ m^2^/s, which is typical for 1 mol/L NaCl and temperatures between 17 and 25 °C [58]. The first performance tests of six test series were used to validate the modelled results. The detailed evaluation of the experiments was published in a previous paper [48].

**Figure 2:**
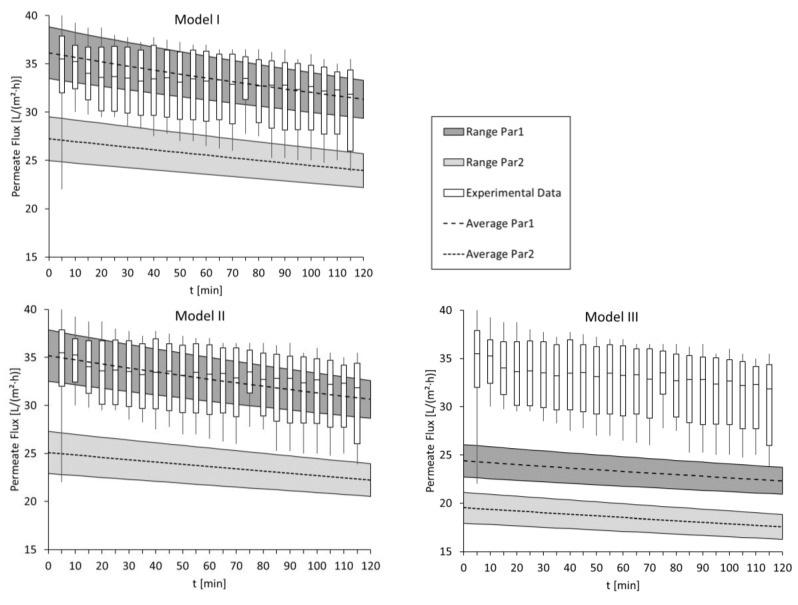
Modelled and experimental permeate fluxes of membrane performance tests. Experimental results are illustrated by boxplots showing the 25% and the 75% quantile as well as the median of six tests. Modelled results are illustrated as broad strips, the width of which is caused by the variation of the diffusion coefficient. The mean value is shown as a dashed line.

Figure 2 shows the experimental and modelled permeate fluxes for the three models (I, II, III) and two membrane parameter sets (Par1, Par2). Experimental results are illustrated by boxplots showing the 25% and the 75% quantile as well as the median of six tests. Modelled results are illustrated as broad strips, the width of which is caused by the variation of the diffusion coefficient. The mean value is shown as a dashed line. The more the modelled results overlap with the experimental results, the better suitable is the applied model and the used membrane parameter set.

It can be seen that Model I and Model II successfully simulated the experiments when Par1 was used since modelled and experimental results overlap almost completely. Model III did not match the experimental permeate fluxes with either membrane parameter set. 

In general, the overlap of experimental and modelled data was higher for Model I and Model II, showing that the performance of Model I and Model II was better than Model III. For the same reason, membrane parameter set Par1, which was determined by FO-only method, is more suitable than Par2, which was determined by RO-FO method. The decrease in permeate flux due to FS and DS recirculation is also well simulated. Membrane fouling is not considered in the modelling but might happen in the experiments. Apparently, no membrane fouling occurred in the performance tests since the decrease of experimental and modelled permeate fluxes are the same. 

Applied model equations assume that the membrane has a porous active layer. The used FO membrane, however, is equipped with a dense active layer. Still, the model equations deliver mostly matching results, proving that the chosen model equations can be applied successfully.

Furthermore, by modelling the membrane performance tests, it was shown that the calibration for assuming the diffusion coefficient is applicable. 

### 3.2. Modelling of Permeate Flux in ALFS Mode for Wastewater Experiments

**Figure 3:**
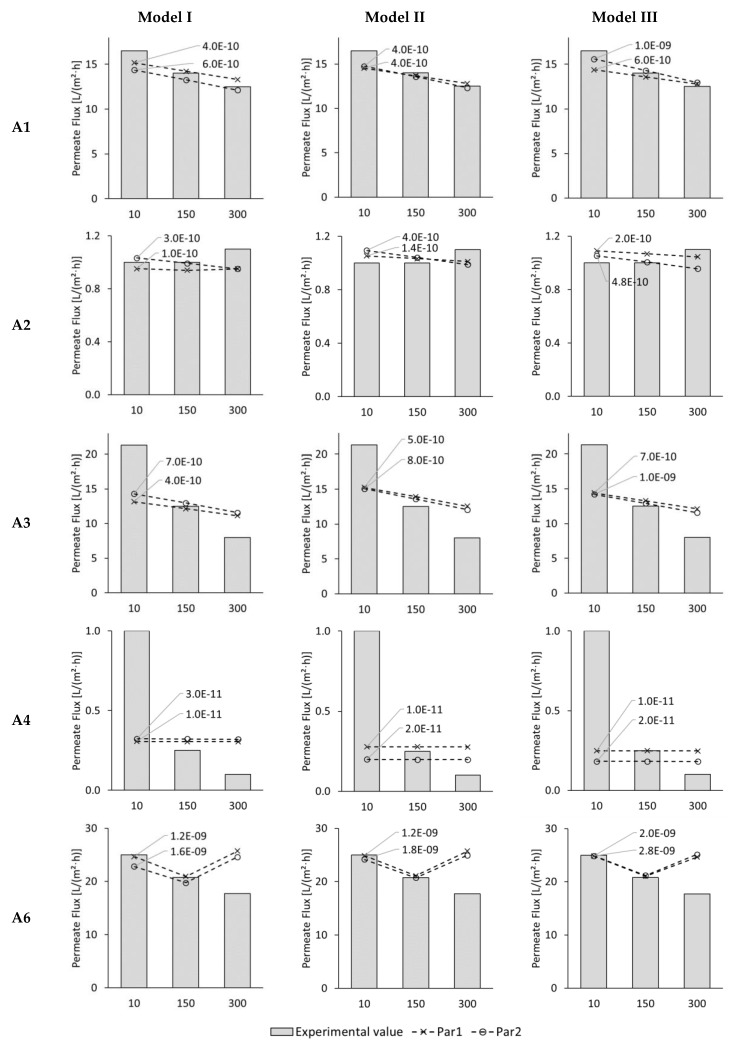
ALFS permeate fluxes of test series A1, A2, A3, A4, and A6 for 10, 150, and 300 min test time from experiments (columns) and Model I, Model II, and Model III with membrane parameter set Par1 (dashed line with crosses) and Par2 (dashed line with circles). Modelled results are shown for best best-fitting diffusion coefficient which is indicated next to the corresponding line.

FO laboratory experiments were conducted with four different automobile wastewaters, deionized water, and 1 mol/L NaCl. In five test series, an ALFS membrane orientation was used. One test lasted 300 min. Previously, the detailed evaluation of the experiments was published elsewhere [48]. In Figure 3, modelled and experimental permeate fluxes are illustrated for the ALFS experiments with automobile wastewater. Permeate fluxes are given exemplarily for 10, 150, and 300 min test time. Experimental values are illustrated with columns. Modelled results for Model I, II, and III are shown as dashed line with crosses when membrane parameter set Par1 was used, and with circles when Par2 was used. Modelling runs were performed with different diffusion coefficients. In Figure 3, only the best fitting modelling run is illustrated and the corresponding diffusion coefficient is indicated next to the dashed line. 

Permeate fluxes differed strongly depending on the utilized FS and DS. For this reason, the axis for the permeate flux was adjusted accordingly. In test series A2 and A4, the experimental permeate fluxes in the experiments were between 0.1 and 1.1 L/(m^2^·h). These low fluxes are due to the low osmotic pressure difference between FS and DS. In test series A1, A3, and A6, the experimental permeate fluxes were between 8.0 and 25.0 L/(m^2^·h). The higher permeate fluxes can be explained by the higher osmotic pressure differences between FS and DS (see Table 1). A detailed evaluation of the experimental results is included in another publication [48].

Due to FS and DS recirculation and permeate flux through the membrane, FS and DS are concentrated and diluted, respectively, during the experiment. For this reason, the osmotic pressure difference between FS and DS also decreases leading to a decreasing permeate flux. This fact is included in the model equations. However, permeate flux in the experiments might additionally decrease because of membrane fouling or temperature changes affecting viscosity as well as diffusion coefficients and mass transfer coefficients. These effects are not considered in the model equations. The slope of the permeate fluxes in Figure 3 illustrates the decreasing permeate flux in the course of FO treatment.

With every model, five test series were modelled with two different parameter sets. Thus, 30 data points are illustrated for each model. The modelled results are described as “good” if the deviation from the experimentally determined value is a maximum of 15% and as “very good” if the deviation is a maximum of 5%.

With Model I, 10 data points were modelled very well, 7 well, and 13 poorly. Model I showed “good” and “very good” results simulating complete test series A1 and test series A2 with Par 2. Furthermore, “very good” results were obtained for the 10-min-flux in test series A2 with Par1, the 150-min-flux in A3 with both Par1 and Par2, the 10- and 150-min-flux in A6 with Par1, and the 150-min-flux in A6 with Par2. “Good” matches were obtained for the 150-min-flux in A2 with Par1 and for the 10-min-flux in A6 with Par2. 

With Modell II, 11 data points were modelled very well, 8 were modelled well, and 11 were modelled poorly. Model II delivered “good” and “very good” results for the complete test series A1 and A2. Further “very good” matches were the 10- and 150-min-flux in A6; further “good” matches were the 150-flux in A3 and the 150-min-flux in A4 with Par1.

With Model III, 13 data points were modelled very well, 5 well, and 12 poorly. Modell III simulated test series A1 and A2 well and very well. Further, “very good” matches were obtained for the 150-min-flux in A3 with Par2 as well as the 10- and 150-min-flux in A6. Another “good” match was found for the 150-min-flux in A3. 

Considering the 30 data points, which were analyzed, Model III delivered the most results labeled as “very good”. However, it delivered one poor result more than Model II. Therefore, Model II and Model III both seem more suitable for modelling the permeate flux because they achieved 19 and 18 good and very good results, respectively.

Test series A1 had permeate fluxes decreasing from 16.5 to 12.5 L/(m^2^·h) and was simulated well by all models. The decreasing permeate flux was well reflected by the models. Since the slope of modelled and experimental values is the same, apparently no fouling occurred during the 300-min-test. Test series A3 had permeate fluxes starting at 21.3 and decreasing to 8.0 L/(m^2^·h). Here, the 150-min-value was well predicted by all models. However, the 10-min-value was underestimated by the models; the 300-min-value overestimated. The slope of the experimental values was steeper than the modelled values. So it is probable that membrane fouling occurred during the experiments. The reason for the high experimental permeate flux in the beginning of the test compared to the modelled results is not clearly identifiable. One reason might be that membrane samples from one manufacturer still differ to some extent and might have different membrane parameters leading to different initial permeate fluxes. 

Test series A2 and A4 had low permeate fluxes below 1.1 L/(m^2^·h). A decreasing permeate flux was modelled for A2 but not measured in the experiment. The constant or even increasing permeate flux that was measured in the experiment might be due to the regular measurement inaccuracy in the experimental set-up, especially, when only low mass changes occurred. Mathematically a higher permeate flux only occurs due to two reasons: A higher osmotic pressure difference or an increasing diffusion coefficient. Reasons for these effects may be rising temperatures or a dilutive ICP in the support layer of the membrane. In regard to A4, a constant permeate flux was modelled but not measured. Again, the 10-min-value was underestimated by the models; the 300-min-value overestimated. Apparently, membrane fouling occurred in A4 and could not be simulated by the models. Due to the very low permeate fluxes, test series A2 and A4 are not ideal for model validation.

Within the experimental test series, A6 is special because a negative reverse solute flux occurred during the experiments. This means that substances from the FS diffused to the DS. Usually, reverse solute flux occurs from draw to feed side in the opposite direction than the permeate flux. None of the applied models was able to consider this effect. Therefore, in order to simulate a permeate flux, the measured reverse solute flux was used as input data instead of calculated reverse solute flux. With this adaption, it was possible to get very good fits between experimental and modelled results for 10 and 150 min test time. The high deviation for the 300-min-value can be explained by the extremely negative experimental reverse solute flux used as input data. Here, problems within the experimental procedure, especially difficulties to measure only small differences in conductivity and changing FS and DS composition, might be the reason for incorrectly measured reverse solute fluxes. Because of the mentioned problems, model validation with test series A6, especially the 300-min-value, should be treated with caution.

In general, it is interesting to see that in most cases the 150-min-value for the permeate flux was well predicted. Regarding the membrane parameters, no parameter set delivered better results than the other. With Par1, 17 “very good” and 11 “good” matches were obtained; with Par2, 17 “very good” and 10 “good” matches were found.

**Figure 4:**
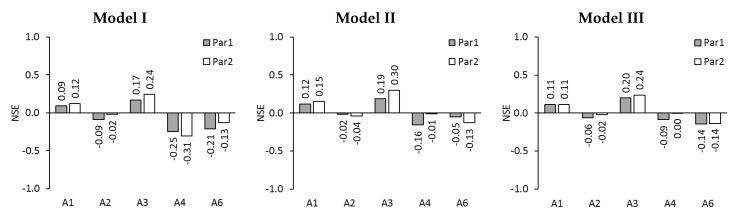
NSE for permeate fluxes of test series A1, A2, A3, A4, and A6 simulated with Model I, Model II, and Model III using membrane parameter set Par1 and Par2.

Figure 4 shows the NSE-values that were obtained with Model I, Model II, and Model III. For Model I, the NSE ranges from –0.31 up to 0.24. NSE-values close to zero and between zero and 1.0 are considered good results. NSE-values between 0 and 1.0 were achieved for test series A1 and A3. NSE-values close to zero were further calculated for A2. Thus, 6 out of 10 NSE-values are good. 

For Model II, the NSE ranges from –0.16 up to 0.30. Good NSE-values between 0 and 1.0 were achieved for test series A1 and A3. NSE-values close to zero were further calculated for A2 as well as A4 with Par2 and A6 with Par1. Altogether, 8 out of 10 NSE-values are good.

For Model III, good NSE-values between 0 and 1.0 were achieved for test series A1 and A3. NSE-values close to zero were further calculated for A2 as well as A4. So 8 out of 10 NSE-values are good.

Since Model II and Model III each delivered 8 good NSE-values, they appear more suitable than Model I, which only delivered 6 good NSE-values. 

### 3.3. Modelling of Reverse Solute Flux in ALFS Mode for Wastewater Experiments

**Figure 5:**
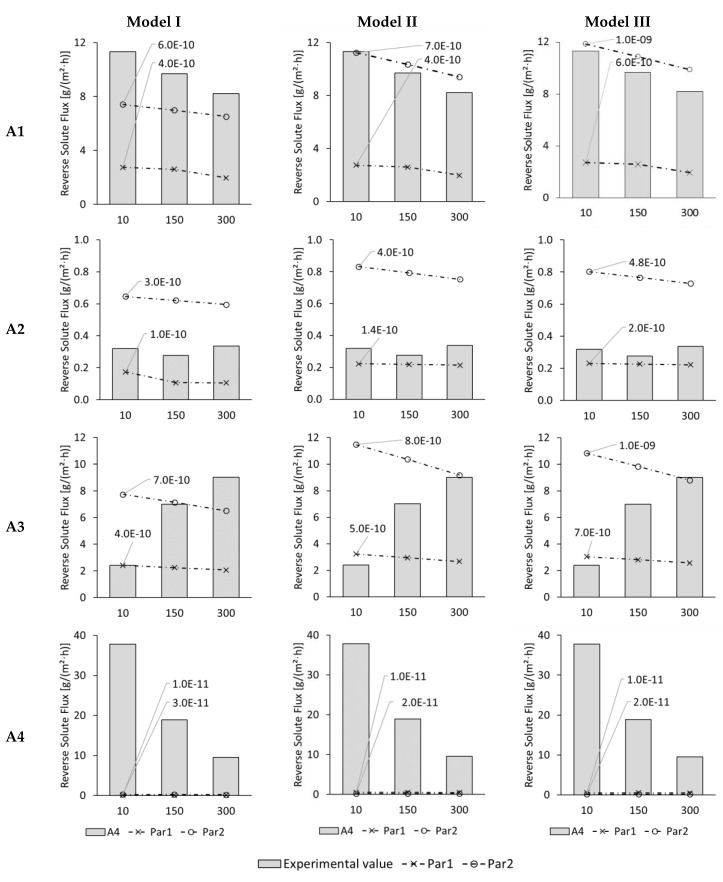
ALFS reverse solute fluxes of test series A1, A2, A3, and A4 for 10, 150, and 300 min test time from experiments (columns) and Model I, Model II, and Model III with membrane parameter set Par1 (dashed line with crosses) and Par2 (dashed line with circles). Modelled results are shown for best-fitting diffusion coefficient which is indicated next to the corresponding line.

In Figure 5, the experimental and modelled reverse solute fluxes (RSF) are illustrated for the test series with automobile wastewaters for 10, 150, and 300 min when the membrane was used in ALFS mode. Modelled results are shown for test series A1, A2, A3, and A4. Here, reverse solute fluxes were between 8.2 and 11.3, between 0.28 and 0.34, between 2.4 and 9.0, and between 37.8 and 9.5 g/(m^2^·h), respectively. No reverse solute flux could be modelled for test series A6 because a negative reverse solute flux occurred here. This means, that substances passed from the FS into the DS, not the other way around as usually expected. All three models were unable to include this phenomenon. 

The driving force for RSF is the concentration gradient which exists between FS and DS. Ideally, a solute flux through the membrane should be prevented by the membrane itself. However, solute flux still occurs with present FO membranes. Due to permeate flux, the concentration gradient decreases in the course of FO treatment and RSF should therefore also decrease. 

In Figure 5, 24 modelled data points are illustrated for each model. As in the previous chapter, when modelled and experimental data points are compared, they are labelled as “very good fit” when the modelled result is within 5% of the experimental result and as “good fit” when both values are in a range of 15%.

Model I only showed two “very good” results in regard to the reverse solute flux. In test series A3, the 10-min-value matched very well when Par1 was used. Furthermore, the 150-min-value in the same test series with Par2 was a “very good” fit. All other 22 data points fitted poorly.

Model II was able to reach two “very good” fits and two “good” fits. The 10-min-value of A1 with Par2 and the 300-min-value in A3 with Par2 fitted “very good”. The 150- and 300-min-value in A1 with Par2 fitted well. 20 data points fitted poorly.

Model III resulted in two “very good” fits and one “good” fit. The 10-min-value in A1 with Par2 as well as the 300-min-value in A3 with Par2 were the “very good” fits. The 150-min-value in A1 with Par2 was the “good” fit. 23 data points were modelled with poor results.

In general, the used modelling approaches were unable to simulate reverse solute flux with satisfying results. However, Model II and Model III delivered slightly better results than Model I.

**Figure 6:**
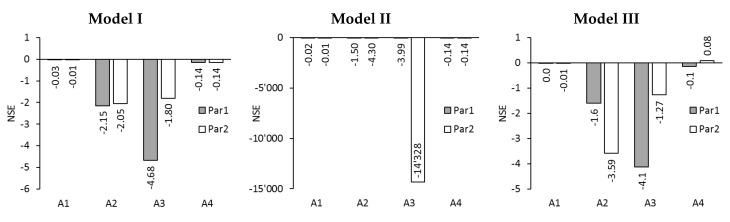
NSE for reverse solute fluxes of test series A1, A2, A3, and A4 simulated with Model I, Model II, and Model III using membrane parameter sets Par1 and Par2.

The NSE- and MSE-values for Model, I, Model II, and Model III are given in Figure 6. NSE-values for Model I are all below zero ranging from –0.01 down to –4.68. That indicates that modelled and experimental reverse solute fluxes did not fit well at all. Only for test series A1, the NSE-values are close to zero with –0.03 and –0.01, which shows that here the modelling was not too bad.

For Model II the NSE-values are also below zero ranging from –0.01 down to –1,4328. Again, only test series A1 was modelled fairly well according to an NSE-value of –0.01 and –0.02. The NSE-value for test series A3 and Par2 was –1,4328 and thus extremely low. The negative NSE-values show that modelled and experimental reverse solute fluxes did not fit well for Model II.

The NSE-values for Model III are between –4.1 and 0.08. NSE-values between 0 and 1.0 were achieved for A1-Par1 andA4-Par2: They were 0.0 and 0.08, respectively. Furthermore, NSE-values just below zero were found for A1-Par2 and A4-Par1: They were –0.01 both times. Out of the three modelling approaches, Model III delivered the best reverse solute fluxes when the NSE-values are taken as evaluation criterion.

RSF was not successfully modelled. For modelling, the electrical conductivity was taken as sole input parameter to minimize analysis effort. It was assumed that the electrical conductivity was caused by NaCl only. In reality, however, RSF strongly depends on the composition of FS and DS because each substance behaves differently in regard to membrane diffusion. This so-called selective diffusion was not considered in the models and might be the reason for discrepancy between modelled and experimental RSF. 

### 3.4. Modelling of Permeate Flux and Reverse Solute Flux in ALDS Mode for Wastewater Experiments

In addition to the five test series that used the ALFS membrane orientation, one test series was performed in ALDS mode. This test series was also modelled using the three modelling approaches described in Section 2.2 and two membrane parameter sets. Permeate fluxes and reverse solute fluxes were determined.

**Figure 7:**
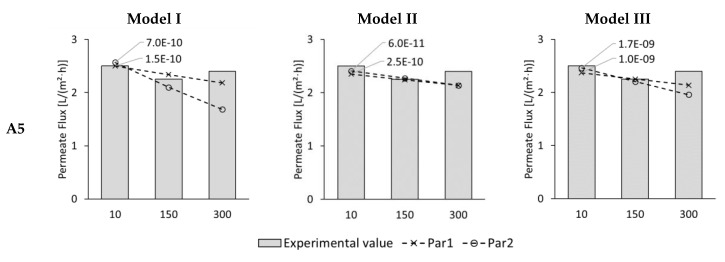
ALDS permeate flux of test series A5 for 10. 150, and 300 min test time from experiments (columns) and Model I, Model II, and Model III with membrane parameter set Par1 (dashed line with crosses) and Par2 (dashed line with circles). Modelled results are shown for best-fitting diffusion coefficient which is indicated next to the corresponding line.

Figure 7 shows the modelled and experimental permeate fluxes for 10, 150, and 300 min test duration. The experimental permeate fluxes were 2.5, 2.3, and 2.4 L/(m^2^·h), respectively. Altogether 6 data points were obtained for each model. Modelled results are labelled as “very good” if the deviation is 5% or less and as “good” if it is 15% or less.

Model I delivered three “very good”, two “good” and only one “poor” result. The “very good” matches were the 10-minute-values as well as the 150-min-value with Par1. The “good” matches were the 150-min-value with Par2 and the 300-min-value with Par1.

Model II resulted in three “very good” and two “good” fits. No “poor” matches occurred with this model. The 10-min-value with Par 2 as well as the 150-min-values were the “very good” fits. The 10-min-value with Par1 and the 300-min-values were the “good” fits.

With Model III four “very good”, two “good”, and one “poor” result was obtained. Here, the 10- and 150-min-values were modelled very well and the 300-min-value with Par1 was modelled well.

If the number of poor matches between experimental and modelled data points are taken as evaluation criterion, all three models showed good permeate flux results with Model II being the best modelling approach.

**Figure 8:**
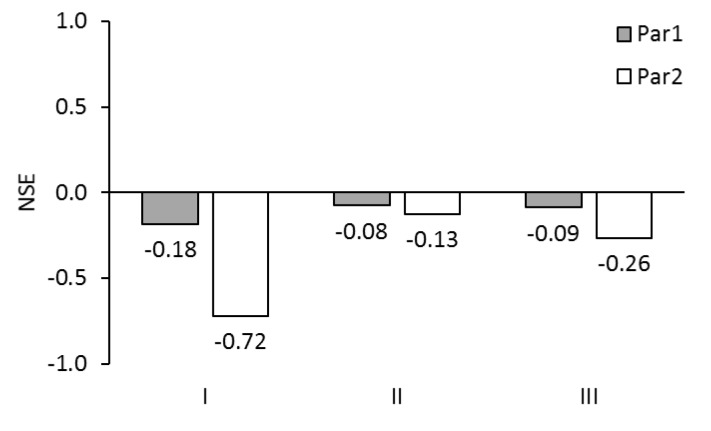
NSE for permeate fluxes of test series A5 (ALDS mode) simulated with Models I, II, and III using membrane parameter sets Par1 and Par2.

NSE-values for the permeate fluxes are illustrated in Figure 8. NSE-values were all below zero ranging from –0.72 up to –0.09. No optimal NSE-values between 0 and 1.0 were achieved. However, NSE-values close to zero were achieved with Model II and Par1 as well as with Model III and Par1: They were –0.08 and –0.09. Taking the NSE-value as evaluation criterion, Model II and Model III combined with Par1 are the most suitable modelling approaches.

**Figure 9:**
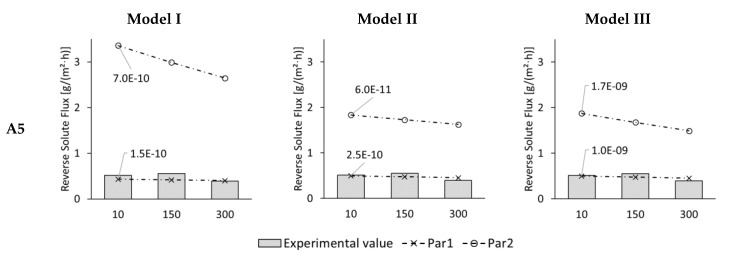
ALDS reverse solute flux of test series A5 for 10. 150, and 300 min test time from experiments (columns) and Model I, Model II, and Model III with membrane parameter set Par1 (dashed line with crosses) and Par2 (dashed line with circles). Modelled results are shown for best-fitting diffusion coefficient which is indicated next to the corresponding line.

In Figure 9, experimental and modelled reverse solute fluxes are illustrated for 10, 150, and 300 min. The experimental reverse solute fluxes were between 0.4 and 0.6 g/(m^2^·h). Six data points were obtained for each model. Modelled results are labelled as “very good” if the deviation is 5% or less and as “good” if it is 15% or less. 

With Model I only one good result was obtained: The 300-min-value with Pa1. All other 5 data points resulted in poor matches, whereby the results with Par1 were closer to the experimental results than with Par2.

One “very good” and one “good” match was found with Model II. Here, again the values with Par1 were better than with Par2. The 10-min-value was the “very good” fit and the 300-min-value was the “good” fit. 

Model III showed similar results as Model II. Again the 10-min-value and the 300-min-value with Par1 were “very good” and “good” matches. All other modelled results matched poorly. The modelled results with Par1 were again closer than with Par2.

With a maximum of two “very good” and “good” matches out of six, reverse solute flux was modelled rather poorly in ALDS-mode. Altogether, reverse solute flux was modelled better with Par1 than with Par2. With Par1 at least 1, 2, and 2 “very good” and “good” matches were achieved for Model I, Model II, and Model III, respectively.

**Figure 10:**
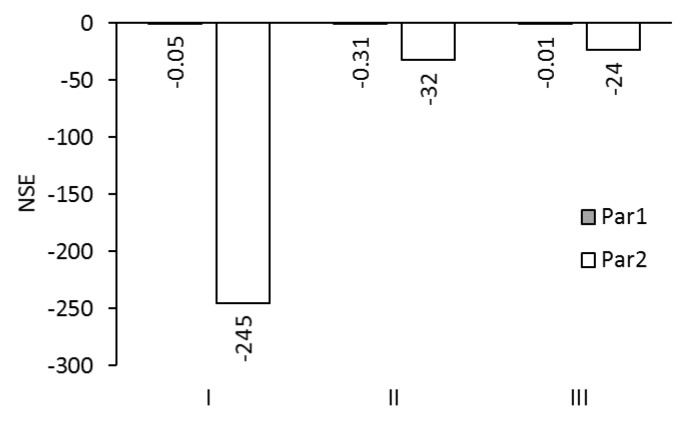
NSE for reverse solute fluxes of test series A5 simulated with Models I, II, and III using membrane parameter sets Par1 and Par2.

The NSE-values for the modelled reverse solute flux with an ALDS membrane orientation are shown in Figure 10. NSE-values were between –245 and –0.01. No optimal NSE-value between 0 and 1.0 occurred. However, NSE-values just below zero resulted from Model I with Par1 and Model III with Par1: They were –0.05 and –0.01, respectively. NSE-values were between –245 and –24 when Par2 was used. Thus, Par2 appears to be unsuitable for reverse solute flux modelling. Par1 appears more suitable because NSE-values were between –0.31 and –0.0.1.

The choice of FS and DS used in test series A6 generated a very low permeate flux. Changes in permeate flux and reverse solute flux were hard to measure within the experiments. For this reason, modelling this one test series in ALDS mode was also error-prone. In order to further validate the ALDS model equations, experiments with FS and DS conditions similar to test series A1, A3, and A6 should have been performed. However, since the experiments were performed separately from the modelling, no further experiment could be conducted. For this reason, a good comparison between ALFS and ALDS modelling was not possible within this study.

## 4. Conclusions

Three different models were implemented in MS Excel to simulate FO lab-scale experiments that were performed with different wastewaters from the automobile industry. The models differed mainly in the extent in which concentration polarization was considered. Permeate flux and reverse solute flux were calculated and compared with the experimental values. Two different membrane parameter sets (water permeability A, solute permeability B, structural parameter S) were used: One that was determined by FO-only test method (Par1) and one that was determined by RO–FO test method (Par2). Five experimental test series used an ALFS membrane orientation, and one used an ALDS membrane orientation.

Within modelling, the automobile experiments, as presented previously by Haupt and Lerch, [48] were simulated. Partly, the permeate fluxes could be well represented others were over- or under-estimated. Here, Model II and Model III showed better results than Model I. There are two possible reasons for the differences between modelled and experimental permeate fluxes: Firstly, fouling effects occurred in the experiments that influenced permeate flux negatively. However, fouling was not considered in the models but could be observed in the experiments. Second, the amount of model input parameters was low. This enabled easy and fast modelling with few analytical expenses. Still, the complex structure and composition of the industrial wastewaters might influence the FO process differently.

The calculation of the reverse solute fluxes showed mostly large deviations compared to the actual measurements. Thus, modifications need to be made to better predict reverse solute flux. One possibility might be the use of more input parameters than just the electrical conductivity and the performance of long-term experiments.

An important parameter of the model equations is the diffusion coefficient. In our study, several modelling runs were performed with different diffusion coefficients. By comparing the results with experimental data, the best fitting diffusion coefficient was chosen for further validation. This approach has produced satisfactory results in our case. However, it is not optimal since the modelling is time-consuming. Moreover, the diffusion coefficient probably changes during FO due to concentration polarization as well as dilution and concentration of DS and FS, respectively. A diffusion coefficient that is concentration-dependant might be implemented for more precise modelling, as studied in depth by D’Haese et al. [59]. Regarding the two membrane parameter sets, neither proved to be better than the other. 

In FO treatment of industrial waters, membrane fouling is a crucial point to be considered. The effect of fouling on permeate fluxes was not part of the model equations. However, the gap between experimental and modelled permeate fluxes allows conclusions to be drawn about the extent of fouling. Thus, the proposed models are a suitable supplement to experiments to predict fouling.

## Figures and Tables

**Table 1:** FS and DS, osmotic pressure difference ∆π, and membrane orientation of the six experimental test series (A1–A6) and the performance tests (P).

Test Series	Feed Solution (FS)	Draw Solution (DS)	∆π [bar]	Membrane Orientation	
				ALFS	ALDS
A1	Cathodic dip painting rinsing water	1 mol/L NaCl	44.8	✓	-
A2	Deionized Water (DI)	Cooling tower circulation water	1.1	✓	-
A3	Paint shop pre-treatment wastewater	1 mol/L NaCl	44.5	✓	-
A4	Deionized Water (DI)	Cathodic dip painting wastewater	2.1	✓	-
A5	Deionized Water (DI)	Cooling tower circulation water	1.1	-	✓
A6	Cathodic dip painting wastewater	1 mol/L NaCl	43.5	✓	-
P	Deionized Water (DI)	1 mol/L NaCl	44.5	✓	-

**Table 2:** Model equations for permeate and reverse solute flux using an ALFS membrane orientation.

Model	Permeate Flux	Reverse Solute Flux	Ref.
I	$J_{w}=\;\frac{D_{DS}}{S}\;ln\left(\frac{B+A\;\pi_{DS}}{B+A\;\left[\pi_{FS}\exp\left(\frac{J_{w}}{k_{FS}}\right)\right]+\;J_{w}}\right)$	$J_{s}=\;B\;\frac{c_{DS}\exp\left(-\frac{J_{w\;}S}{D_{DS}}\right)-\;c_{FS}\exp\left(\frac{J_{w}}{k_{FS}}\right)}{1+\frac{B}{J_{w}}\left[exp\left(\frac{J_{w}}{k_{F}}\right)-\exp\left(-\frac{J_{w}S}{D_{DS}}\right)\right]}$	[16] mod., [46]
II_ALFS_	$J_{w}=\;A\;\frac{\pi_{DS}\exp\left(-\frac{J_{w\;}S}{D_{DS}}\right)-\;\pi_{FS}\exp\left(\frac{J_{w}}{k_{FS}}\right)}{1+\frac{B}{J_{w}}\left[exp\left(\frac{J_{w}}{k_{FS}}\right)-\exp\left(-\frac{J_{w}S}{D_{DS}}\right)\right]}$		
III	$J_{w}=\;A\;\frac{\pi_{DS}\exp\left[-\;J_{w}\left(\frac{1}{k_{DS}}+\frac{S}{D_{DS}}\right)\right]-\;\pi_{FS}\exp\left(\frac{J_{w}}{k_{FS}}\right)}{1+\frac{B}{J_{w}}\left[exp\left(\frac{J_{w}}{k_{FS}}\right)-\exp\left[-\;J_{w}\left(\frac{1}{k_{DS}}+\frac{S}{D_{DS}}\right)\right]\right]}$	$J_{s}=\;B\;\frac{c_{DS}\exp\left[-\;J_{w}\left(\frac{1}{k_{DS}}+\frac{S}{D_{DS}}\right)\right]-\;c_{FS}\exp\left(\frac{J_{w}}{k_{FS}}\right)}{1+\frac{B}{J_{w}}\left[exp\left(\frac{J_{w}}{k_{FS}}\right)-\exp\left[-\;J_{w}\left(\frac{1}{k_{DS}}+\frac{S}{D_{DS}}\right)\right]\right]}$	[47]

water permeability A, solute permeability B, structural parameter S, osmotic pressure π, concentration c, diffusion coefficient D, mass transfer coefficient k, feed solution indexed FS, draw solution indexed DS.

**Table 3:** Model equations for permeate and reverse solute flux using an ALDS membrane orientation.

Model	Permeate Flux	Reverse Solute Flux	Ref.
I	$J_{w}=\;\frac{D_{FS}}{S}\;ln\left(\frac{B+A\;\pi_{DS\;}\exp\left[-\frac{J_{w}}{k}\right]\;-J_{w}}{B+A\;\pi_{FS}}\right)$	$J_{s}=\;B\;\frac{c_{DS}\exp\left(-\frac{J_{w}}{k_{DS}}\right)-\;\pi_{FS}\exp\left(\frac{J_{w}S}{D_{FS}}\right)}{1+\frac{B}{J_{w}}\left[\exp\left(\frac{J_{w}S}{D_{DS}}\right)-exp\left(-\frac{J_{w}}{k_{DS}}\right)\right]}$	[16] mod., [44]
II_ALDS_	$J_{w}=\;A\;\frac{\pi_{DS}\exp\left(-\frac{J_{w}}{k_{DS}}\right)-\;\pi_{FS}\exp\left(\frac{J_{w}S}{D_{FS}}\right)}{1+\frac{B}{J_{w}}\left[\exp\left(\frac{J_{w}S}{D}\right)-exp\left(-\frac{J_{w}}{k_{DS}}\right)\right]}$		
III	$J_{w}=\;A\;\frac{\pi_{DS}\exp\left(-\frac{J_{w}}{k_{DS}}\right)-\;\pi_{FS}\exp\left[J_{w}\left(\frac{1}{k_{FS}}+\frac{S}{D_{FS}}\right)\right]}{1+\frac{B}{J_{w}}\left[\exp\left[\;J_{w}\left(\frac{1}{k_{FS}}+\frac{S}{D_{FS}}\right)\right]-exp\left(-\frac{J_{w}}{k_{DS}}\right)\right]}$	$J_{s}=\;B\;\frac{c_{DS}\exp\left(-\frac{J_{w}}{k_{DS}}\right)-\;c_{FS}\exp\left[J_{w}\left(\frac{1}{k_{FS}}+\frac{S}{D_{FS}}\right)\right]}{1+\frac{B}{J_{w}}\left[\exp\left[\;J_{w}\left(\frac{1}{k_{FS}}+\frac{S}{D_{FS}}\right)\right]-exp\left(-\frac{J_{w}}{k_{DS}}\right)\right]}$	[47]

water permeability A, solute permeability B, structural parameter S, osmotic pressure π, concentration c, diffusion coefficient D, mass transfer coefficient k, feed solution indexed FS, draw solution indexed DS.

**Table 4:** Membrane parameters used for modelling.

Membrane Parameter Set	Water Permeability A [L/(m^2^ h bar)]	Solute Permeability B [L/(m^2^ h)]	Structural Parameter S [10^-6^ m]	Method	Lit.
Par1	5.36	0.95	266	FO-only	[49]
Par2	8.9 ± 0.14	5.68 ± 0.14	466	RO–FO	[50]

## Nomenclature

A	Water permeability [L/(m^2^ h bar)]
A_CS_	Cross-section area [m^2^]
B	Solute permeability [L/(m^2^ h)]
c	Concentration [mol/L]
c_osm_	Osmolality [osmol/kg]
d_h_	Hydraulic diameter [m]
D	Diffusion coefficient of solution [m^2^/s]
J	Flux
J_S_	Reverse solute flux [g/(m^2^·h)]
J_W_	Permeate flux [L/(m^2^·h)]
k	Mass transfer coefficient from bulk solution to membrane interface [m/s]
L	Flow channel length [m]
l_u_	Wetted perimeter [m]
MSE	Mean square error
NSE	Nash-Sutcliffe efficiency [-]
R	Universal gas constant [J/(K·mol)]
Re	Reynolds number [-]
S	Structural parameter [m]
Sc	Schmidt number [-]
Sh	Sherwood number [-]
T	Temperature [K]
v	Fluid velocity [m/s]
*Greeks*	
β	Concentration [g/L]
ρ	Density [kg/m^3^]
Δ	Difference
κ	Electrical conductivity [µS/cm or mS/cm]
η	Dynamic viscosity [Pa·s]
π	Osmotic pressure [bar]
ϑ	Temperature [°C]
*Subscripts*	
DS	Draw solution
exp	Experimental value
FS	Feed solution
mod	Modelled value
N	Number of values
t	Time
